# Antioxidants and Male Infertility

**DOI:** 10.3390/antiox11061152

**Published:** 2022-06-12

**Authors:** Ricardo Silva, David F. Carrageta, Marco G. Alves, Branca M. Silva, Pedro F. Oliveira

**Affiliations:** 1 LAQV-REQUIMTE and Department of Chemistry, University of Aveiro, Campus Universitário de Santiago, 3810-193 Aveiro, Portugal; rsilva11@ua.pt; 2 Clinical and Experimental Endocrinology, UMIB—Unit for Multidisciplinary Research in Biomedicine, ICBAS—School of Medicine and Biomedical Sciences, University of Porto, Rua Jorge Viterbo Ferreira 228, 4050-313 Porto, Portugal; davidcarrageta@gmail.com (D.F.C.); alvesmarc@gmail.com (M.G.A.); 3 Laboratory for Integrative and Translational Research in Population Health (ITR), University of Porto, 4050-313 Porto, Portugal; 4 Biotechnology of Animal and Human Reproduction (TechnoSperm), Institute of Food and Agricultural Technology, University of Girona, ES-17003 Girona, Spain; 5 Unit of Cell Biology, Department of Biology, Faculty of Sciences, University of Girona, ES-17003 Girona, Spain; 6 Faculty of Health Sciences, University of Beira Interior, Av. Infante D. Henrique, 6200-506 Covilhã, Portugal

Oxidative stress has been associated with decreased sperm quality and male infertility. While physiological levels of reactive oxygen species (ROS) are required for male fertility, particularly for acrosome reaction and capacitation, higher levels of ROS lead to alterations in sperm quality, such as motility, viability, and DNA damage [1]. High oxidative stress status has multifactorial etiology as it can occur as a consequence of inflammation and several diseases, including metabolic disorders, or their treatment, such as those related to cancer and autoimmune diseases [2]. In addition, oxidative stress can arise from assisted reproductive techniques (ART) procedures, which may compromise fertility treatment outcomes [3]. Most of the therapeutic approaches to minimize the damage of an increase in ROS are based on the use of synthetic or natural antioxidants derived from natural sources. Examples of these antioxidants and their effects against ROS are reported in several studies included in our Special Issue “Antioxidants and Male infertility” [4,5,6,7].

Sperm cryopreservation, a conventional technique widely used for long-term sperm storage, is known to induce cryodamage and consequent decline in the quality of sperm. Although sperm cryodamage is multifactorial, oxidative stress plays a major role. To ameliorate sperm oxidative damage during cryopreservation, Ponchia et al. studied the effects of the natural antioxidant myo-inositol. The results indicate that myo-inositol, particularly when added after thawing, improved sperm quality and decreased oxidative stress biomarkers. Taken together, the addition of myo-inositol to cryopreservation media and procedures has the potential to improve cryopreservation outcomes [4]. Pini et al. also studied the protective effects of a natural antioxidant against oxidative stress-related DNA damage in spermatozoa [5]. In this study, the authors showed that the administration of acai based capsules, rich in phenolic compounds, for 74 days led to a decrease in sperm DNA fragmentation with a success rate of 68.6%, even in men with poor sperm parameters or very high DNA fragmentation at baseline.

Oxidative stress is also a consequence of autoimmune diseases and cancer treatments, which result in male infertility. Cyclophosphamide, a chemotherapy agent, is known to induce infertility due to the actions of its metabolite acrolein, which causes oxidative stress and apoptosis. Fusco et al. studied the protective effects of Hidrox^®^, an aqueous extract of olive oil containing 40%–50% of the natural antioxidant hydroxytyrosol, a phenolic phytochemical compound, against cyclophosphamide. The authors showed that the oral administration of Hidrox^®^ protected against cyclophosphamide-induced decreased sperm parameters through the modulation of the physiological antioxidant defenses and protection against oxidative damage [6].

Advanced paternal age is also associated with increased ROS production and poorer fertilization and pregnancy rates. Nikitaras et al. studied the effects of the addition of idebenone, a cell-permeable antioxidant, to sperm wash media in spermatozoa of both older men (>40 years) and CBAF1 mice (12–18 months). The authors reported that idebenone reduced ROS levels in both old men and mice, which improved fertilization rates, embryo quality, and implantation rates after in vitro fertilization (IVF). In addition, the supplementation of idebenone improved blastocyst cryosurvival rates [7].

Two research studies included in our Special Issue “Antioxidants and Male Infertility” further shed some light on the mechanisms behind oxidative stress-related male infertility. In an observational and prospective study including 52 men, Berby et al. reported that infertile men had significantly increased levels of cytoplasmic ROS and chromatin condensation defects as well as a higher mean number of telomere signals per spermatozoon in comparison with healthy men [8]. Kongmanas et al. observed that increased ROS production occurs due to seminolipid accumulation in Sertoli cells from old lysosomal arylsulfatase A (ARSA) knockout mice. The accumulation of seminolipid is a lysosomal storage disorder, which results in increased superoxide anion radical and hydrogen peroxide production by Sertoli cells. The increased ROS production results in the apoptosis of Sertoli cells and oxidative damage in testicular germ cells, which impaired spermatogenesis [9].

The protective role of antioxidants against the exposure to endocrine disruptors and consequent increased oxidative stress is also highlighted by two reviews included in our Special Issue. Heavy metals are endocrine disruptors that have been associated with increased oxidative stress and male infertility. López-Botella et al. highlighted the association between exposure to heavy metals and poorer sperm quality through the altered reproductive hormone levels and impaired spermatogenesis [10]. Santiago et al. reviewed the deleterious effects of Bisphenol A, an endocrine disruptor commonly present in epoxy resins and polycarbonate plastics, in male fertility [11]. In this review, the authors highlighted the impairment of the hypothalamic-pituitary-gonadal axis and the increased oxidative stress caused by bisphenol A, which can be prevented or reverted through the use of antioxidants.

Seminal plasma and female reproductive tract antioxidants can be fundamental to preventing alterations in sperm quality by ROS, which was discussed by Ribeiro et al. [12]. In this review, the authors discussed the antioxidant components present in the seminal and female reproductive tract fluids, as well as their association with fertility outcomes. The importance of the antioxidant capacity for fertilization was also highlighted. The extensive use of antioxidants, however, can also be detrimental to spermatozoa and male fertility. Symeonidis et al. deeply discussed that an antioxidant balance is necessary for fertility [13]. In this review, the authors discussed evidence that indiscriminate consumption may lead to sperm damage through a reductive-stress-induced state, a phenomenon addressed as “the antioxidant paradox”. In addition, the potential measurement of oxidation-reduction potential (ORP) as an ancillary tool to basic semen analysis was discussed, including the novel Male infertility Oxidative System (MiOXSYS^®^). According to the authors, this method offers a quick, reliable, and reproducible ORP quantification.

Oxidative stress can damage sperm at several degrees. The effects of oxidative stress on the sperm genome, however, do not only comprise DNA fragmentation. Ghaleno et al. discussed DNA base oxidation and the consequent genetic and epigenetic alterations [14]. In this review, the authors highlighted that guanine and adenosine residues are particularly sensitive to oxidative damage, which can compromise embryo genetic material independently of sperm DNA fragmentation. As spermatozoa lack DNA repair systems, de novo mutation transmission to the embryo is increased if the zygote is unable to repair the oxidized bases.

Varicocele is an abnormal dilation of the pampiniform plexus which is associated with male infertility. Antioxidant supplementation is often advised for these patients. Pyrgidis et al. conducted a systematic review, which included 14 studies, on the effect of antioxidant supplementation on varicocele patients [15]. These authors observed that antioxidant supplementation did not improve pregnancy rates and semen parameters in varicocele cases. In addition, these authors highlighted that most studies had methodological issues and raised awareness that further research in this field is necessary.

Natural and synthetic antioxidants are widely used to address male infertility, but more studies are needed to understand the specificity and effectivity of these compounds. It is necessary to raise awareness against the indiscriminate use of antioxidants, which may go beyond the beneficial therapeutic effects. As male infertility cases tend to increase and more couples seek fertility counseling worldwide, “Antioxidants and Male Infertility” is a timeless and crucial research topic, as supported by novel and reviewed data analyzed in the articles included in this Special Issue.
